# Impact of Different Onboarding Strategies on Low Adoption and Engagement With a Self-Monitoring and Management App for Chronic Musculoskeletal Pain: Prospective Study

**DOI:** 10.2196/78827

**Published:** 2026-03-30

**Authors:** Cinja Nadana Koller, Marc Blanchard, Johanna Mettler, Tiffany Prétat, Pedro Ming Azevedo, Thomas Hügle

**Affiliations:** 1 Lausanne University Hospital and University of Lausanne Lausanne Switzerland

**Keywords:** onboarding strategies, adoption, mHealth, adherence, engagement, chronic musculoskeletal pain

## Abstract

**Background:**

Suboptimal adoption and engagement rates of digital health applications present challenges to their effectiveness, particularly in chronic disease management such as fibromyalgia. Up to half of patients do not download the prescribed digital health applications or actively engage with them, making effective onboarding a critical opportunity for improvement.

**Objective:**

We aimed to investigate the impact of 3 different patient onboarding strategies on adoption, adherence, and engagement with a digital health application for the symptom management of chronic pain syndromes.

**Methods:**

We conducted a 4-week nonrandomized prospective study comprising patients (aged ≥18 years) with chronic musculoskeletal pain who fulfilled the fibromyalgia criteria and were using a new self-monitoring and management application after onboarding. The pain organizer and companion system consists of symptom reporting, symptom monitoring, and an advice and exercise section. Participants in group 1 (standard) received an email-based onboarding with detailed instructions on application download, registration, use, usability data capture, data security, and study procedures without any in-person assistance. Group 2 (video) received the same email plus a video tutorial explaining the application’s use also without any in-person assistance. Group 3 (assisted) underwent an enhanced in-person onboarding, which included hands-on support for application setup and guided instructions from a health care professional. Primary outcomes included adoption, engagement, and adherence, measured respectively by successful application download with at least 1 log-in, cumulative activations, and connection at least once per week over 4 weeks.

**Results:**

A total of 48 patients were recruited (mean age 45.7, SD 12.2; range 19-79 years). In the assisted group, all 15 participants in the assisted onboarding group downloaded the application compared to 63% (10/16) in the standard group and 77% (13/17) in the video group. A significant difference in adoption between the in-person onboarding group (group 3) and the remote groups (groups 1 and 2) with *P*=.009 was observed. Overall engagement, measured by cumulative app activation, ranged between 0 and 28 log-ins, with no significant difference (*P*=.18) in mean log-ins across the 3 groups. Overall adherence was low with 27% (13/48) of participants meeting adherence criteria and a retention rate of 46% (22/48) at week 4. Responses to the System Usability Scale survey were limited to 8 responses but achieved a score of 70.31, indicating good usability of the application.

**Conclusions:**

Unassisted downloading and account creation might be barriers to adopting a digital health app among patients with chronic musculoskeletal pain.

## Introduction

Digital health solutions, including mobile applications and digital therapeutics, have emerged as promising tools in modern health care delivery. A growing body of evidence suggests that digital health solutions, when effectively adopted and integrated into patient care, can result in positive health outcomes. These technologies offer the potential to enhance patient engagement, improve treatment adherence, and empower individuals to actively manage their health, particularly in the context of chronic conditions [1-3]. However, the active use of digital interventions influences their effectiveness. It was found that increased user engagement with digital interventions is linked to greater improvements in outcomes [4]. However, despite the increasing availability and potential benefits of digital health solutions, achieving high levels of adoption and engagement remains a significant challenge [5-7]. Studies consistently report that only about half of prescribed digital health solutions are downloaded by patients, leading to low use rates and missed opportunities for improved health management [8,9]. This discrepancy between the potential benefits of digital health solutions and their limited adoption and engagement rates raises critical questions about the factors influencing adoption and the potential strategies for enhancing adoption [10]. Implementation science examines whether an intervention is delivered as intended and investigates strategies to enhance its integration into real-world settings, using outcomes such as adoption, engagement, and retention to assess implementation success [11].

Increased adoption rates not only facilitate the effective use of these technologies but also hold the potential to positively impact patient outcomes, reduce health care costs, and improve overall health care delivery [3,12]. Among the various factors affecting adoption and engagement, the onboarding process plays a pivotal role [6]. Onboarding refers to the initial introduction of a patient to a digital health solution including, for example, downloading, registering, and device pairing [13,14]. A major reason for the low adoption of mobile health (mHealth) applications is concerns about data security and privacy [15,16]. Another barrier to adoption is the lack of support from physicians [17]. However, according to Dahlhausen et al [18], health care professionals (HCPs) hold significant potential to enhance adherence to digital therapeutics. Moreover, Hernandez-Ramos et al [14] found low self-reported digital health literacy among study participants reporting difficulties with new tools such as mHealth apps. They addressed this issue by providing onboarding support (eg, downloading and installing the application) to digital health platforms.

Therefore, onboarding serves as a critical opportunity to engage and motivate patients, establish trust, and familiarize them with the functionalities and benefits of the technology. A well-designed onboarding process can potentially address patient concerns, mitigate barriers to adoption, and optimize the user experience, ultimately increasing the likelihood of sustained engagement and use of the digital health solution. Given the importance of adoption and the critical role of onboarding strategies, it is essential to explore how different onboarding approaches influence adoption rates, user engagement, and retention of digital health solutions. Despite the importance of onboarding, many studies focus only on engagement strategies or barriers to adoption after the initiation of the digital intervention [10,15,16,19-24]. In this study, we aim to address this gap by investigating the effect of patient onboarding strategies, used as an implementation strategy, on the adoption, engagement, and retention of a digital health solution for chronic conditions, specifically for the management of chronic musculoskeletal pain. By evaluating the impact of tailored onboarding approaches compared with a standard onboarding process, we seek to identify effective strategies that can increase adoption, enhance retention, and optimize the overall benefits of digital health interventions.

## Methods

### Study Participants

The study included individuals who had been diagnosed with chronic musculoskeletal pain resistant to conventional treatment. Participants had to be aged ≥18 years and have the capability to use a mobile app–based intervention. Additionally, participants needed to possess an email account and a smartphone capable of downloading and using the study-specific self-monitoring and self-management application. Individuals with insufficient proficiency in French to provide informed consent or complete study procedures were excluded. Participants were recruited from the University Hospital Lausanne after they underwent a 2-week in-house multimodal pain program due to being refractory to outpatient treatment. These patients typically present with severe pain as well as high levels of anxiety, depression, catastrophizing, and cognitive impairments, including deficits in attention and memory.

### Ethical Considerations

Ethics approval was obtained from the Commission Cantonale d’éthique de la Recherche sur l’être Humain (CER-VD 2021-01680). Informed written consent was given by all participants prior to their inclusion in the study. The study was conducted ethically in accordance with the World Medical Association Declaration of Helsinki. Data were deidentified to protect participant privacy and maintain confidentiality. Participants did not receive any compensation for taking part in this study.

### Study Design

**Figure 1:**
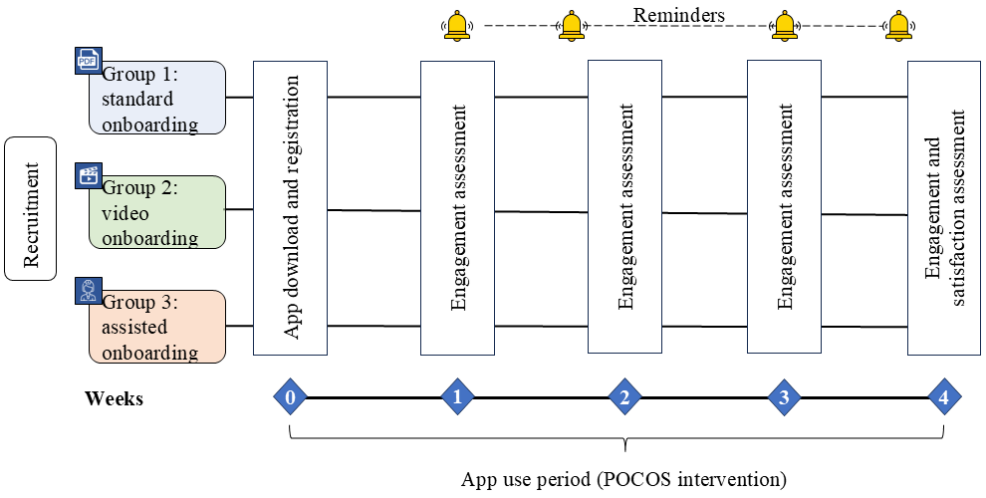
Study design. Patient-reported outcomes were collected through the pain organizer and companion system (POCOS) app. Engagement was assessed through usability data, and satisfaction was assessed using the System Usability Scale. Reminders to use the POCOS app were delivered via SMS text messages.

This comparative, nonrandomized 4-week study examined the effect of different onboarding strategies on the adoption of an mHealth application and retention of use in patients with chronic musculoskeletal pain (Figure 1). Eligible participants were enrolled sequentially into the following three groups (each representing a different onboarding strategy):

1. Group 1 (standard onboarding group): participants in this group received a standard onboarding process that was delivered entirely remotely via email. This onboarding included step-by-step instructions for downloading and registering an account, information on application use and usability data collection, details regarding data security, an overview of study procedures related to the self-monitoring and self-management application, and a concise summary of the application’s core features and functionalities. The manual can be accessed in Multimedia Appendix 1 (in French).
2. Group 2 (video onboarding group): participants in this group underwent the same onboarding process as group 1 but were also provided with an onboarding video. This video, of 3 minutes duration, walked the participants through a comprehensive demonstration of how to use various features and functionalities of the application. The video can be accessed [25].
3. Group 3 (assisted onboarding group): participants in this group received onboarding assistance from a dedicated study HCP via a 30-minute in-person session at the University Hospital Lausanne. The HCP guided each participant individually through the onboarding process and assisted in downloading, registering, logging in, and entering initial data into the application. Furthermore, the HCP provided personalized support and explanations, addressing any questions or concerns raised by the participants during the onboarding session. To minimize bias, the HCP followed a script.

Participants in all 3 groups had access to the same user manual to guide them through application use. The onboarding procedures in groups 1 and 2 were fully remote, while group 3 was in person. Blinding of HCPs and participants was not feasible due to the nature of the intervention.

All participants had the possibility to reach out for support via email or phone for assistance with any step during the study time.

Adoption and engagement are well-documented challenges in mHealth applications, with several studies identifying key barriers and influencing factors. The literature emphasizes the importance of reminders, user-friendly and robust technical design, data privacy and security, tailored content, and personal support [19,20,26]. To address these factors and enhance adherence and engagement, we incorporated them into the study design alongside the specific onboarding strategies. All participants received weekly SMS text message reminders to engage with the pain organizer and companion system (POCOS) application and to provide usability data. To mitigate concerns around data privacy and security, information on secure data handling was explicitly addressed during onboarding. The POCOS application was co-designed with patients and HCPs and features a user-friendly interface with reliable technical performance [27].

### POCOS User Interface

**Figure 2:**
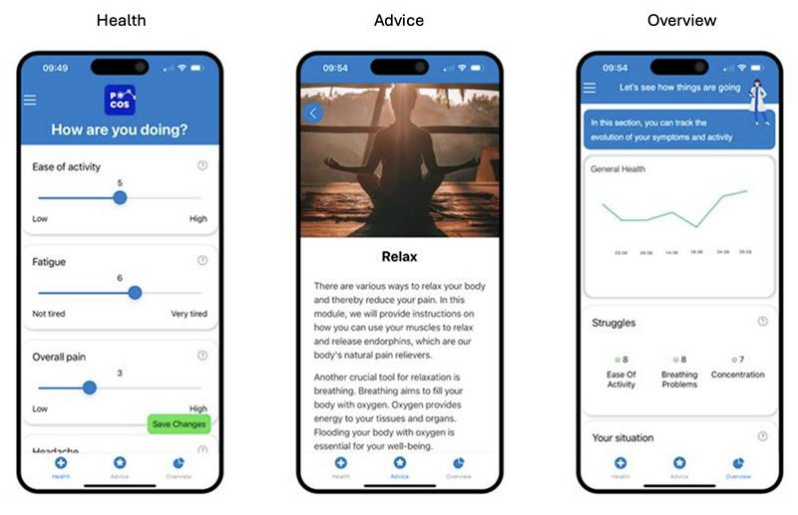
Pain organizer and companion system user interface. Navigation is provided via the bottom menu with 3 main sections: Health, Advice, and Overview.

POCOS is a self-monitoring and management application, which was tested in a previous study [27,28]. This application consists of 3 components: symptom reporting, symptom monitoring, and an advice and exercise section (Figure 2). Moreover, the content can be tailored by patients, as they can freely navigate through the application. Following the onboarding phase, participants in all 3 groups were asked to use POCOS 2 to 3 times a week for 1 month. During this period, patient-reported outcomes related to chronic pain (fatigue, concentration, sleep quality, memory, pain, ease of activity, and anxiety) were asked to be reported by patients via the application at baseline, after 2 weeks, and after 4 weeks of using the application. At the end of the study, participants received a link via text message to fill out a System Usability Scale (SUS) survey.

### Outcomes Measures

#### Primary Outcomes

The primary outcomes of this study were adoption, adherence, and engagement with the application. Adoption was defined as the uptake or intention to try or use a new intervention [29]. The primary measure was the adoption rate of the self-monitoring and self-management application among participants. Adoption rates were assessed by calculating the percentage of participants who successfully downloaded the application and logged in at least once.

Adherence was measured as the percentage of participants who continued using the application after the initial download and remained engaged with at least 1 connection per week throughout the 1-month period.

Engagement was assessed over the 4-week period using the number of log-ins and time spent with the application, two commonly used application engagement metrics [30].

#### Secondary Outcome

The secondary outcome of this study was patient satisfaction, assessed at the end of the study using the SUS survey. In the survey, participants were asked how they liked the application and how satisfied they were with the onboarding support that they had received.

### Data Collection and Management

Data collection for this study was fully decentralized and involved a combination of participant-reported data and application-collected use data. Participants were provided with secure log-in credentials to access the self-monitoring and self-management application. All data were coded (deidentified) and stored in a secure, password-protected database. The application use and engagement metrics, such as account registration and frequency of application use, were recorded. These engagement measures were obtained passively via the database or actively through screenshots of application use that had to be sent biweekly by participants to the research team. At the end of the study, participants received a link for a follow-up SUS survey, and all responses were fully anonymized.

### Statistical Analysis

Descriptive statistics were calculated for all variables, including means and SDs for continuous variables and frequencies and percentages for categorical variables. Differences in continuous outcomes between the 3 onboarding groups were assessed using the Kruskal-Wallis *H* test, as the data did not meet the assumption of normality. Group differences and associations between categorical variables (adoption and adherence) were assessed using the chi-square test or Fisher exact test (if <5 observations per category). Generalized linear modeling (linear or logistic, as appropriate) was used to examine the associations between user characteristics and engagement. To analyze survival rate and time among the different groups, we used Kaplan-Meier survival functions with a log-rank (Mantel-Cox) test. Two-sided *P* values <.05 were considered statistically significant, and 95% CIs were used. All analyses were performed with SPSS software (version 29.0.2.0; IBM Corp). We analyzed with an intention-to-treat approach.

## Results

### Overview

A total of 48 patients with chronic musculoskeletal pain were recruited from 2023 to 2025 and sequentially distributed into 3 groups: 16 (33%; standard), 17 (35%; video), and 15 (31%; assisted) participants per group. Mean age was 45.7 (SD 12.2) years, and 40 (83%) participants were female. Overall, 39 (81%) participants had depression, 12 (25%) had anxiety, and 18 (38%) were opioid users. All participants fulfilled the fibromyalgia criteria. No clinically meaningful differences in baseline characteristics were observed. Detailed clinical information per group is shown in Table 1.

**Table 1:** Clinical and demographic characteristics of the study participants (N=48).

Characteristics				Standard onboarding^a^		Video onboarding		Assisted onboarding
**Comorbidities, n (%)**								
	Fibromyalgia criteria fulfilled			16 (100)		17 (100)		15 (100)
	**Depression**							
		Yes	13 (81)		14 (82)		12 (80)	
		No	2 (13)		3 (18)		3 (20)	
	**Anxiety**							
		Yes	2 (12)		6 (35)		4 (27)	
		No	13 (81)		11 (65)		11 (73)	
	**Opioid use**							
		Yes	5 (31)		6 (35)		7 (47)	
		No	11 (69)		11 (65)		8 (53)	
**Sex, n (%)**								
	Female			16 (100)		13 (76)		11 (73)
	Male			0 (0)		4 (24)		4 (27)
Age (y), mean (SD)				46.3 (14)		45.9 (12)		44.9 (10)

^a^n=1 is missing for depression and anxiety in the standard onboarding group.

### Adoption: Downloads and First Engagement

**Figure 3:**
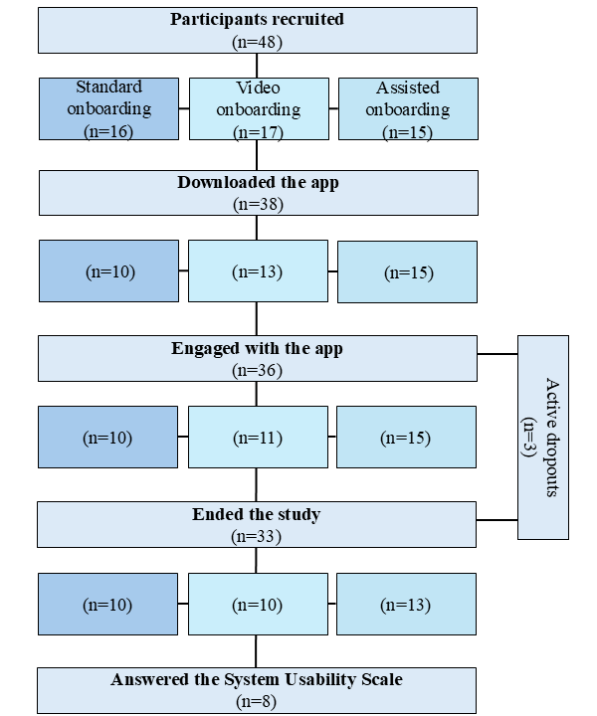
Participant flow per onboarding type.

Overall, 81% (39/48) of participants downloaded the application after they received the instructions. All 15 participants in the assisted onboarding group downloaded and registered the application, while 63% (10/16) of participants registered an account in the standard onboarding without assistance. In the video group, 76% (13/17) of participants downloaded and registered an account. In total, 24% (4/17) of participants in the video group contacted the research team for assistance as they struggled with completing the onboarding independently. Support was given via phone or email by the research team. Of these 15 assisted participants, 1 (7%) had additional help from a relative to complete the downloading. Of them, 3 (20%) participants successfully managed to create their accounts, while 1 (7%) was lost in follow-up. Overall, 75% (36/48) of participants adopted the application, meaning that they successfully downloaded and tested the application at least once. About 69% (33/48) completed the study, meaning that they did not actively drop out of the study. Figure 3 shows the flow of participants in the different groups.

**Figure 4:**
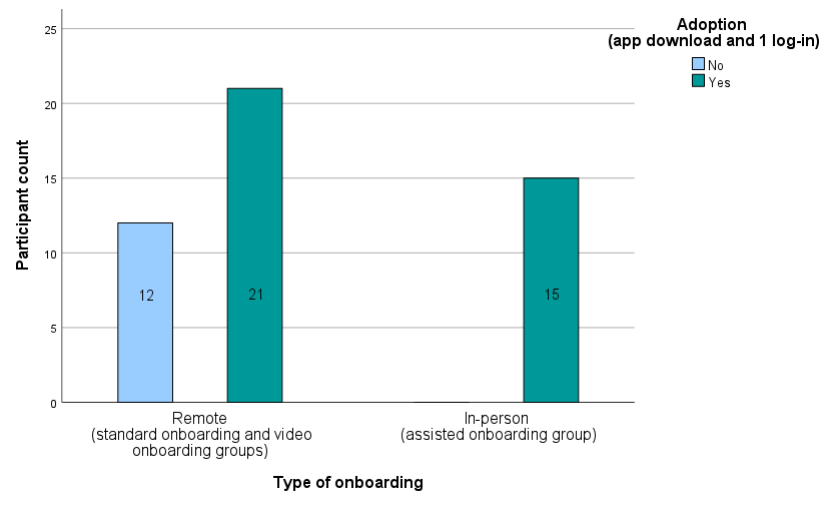
Adoption of the application in the remote onboarding vs the in-person onboarding group.

In a combined analysis of remote onboarding (standard and video onboarding) vs assisted in-person onboarding, adoption was significantly higher in the in-person onboarding group (*df*=1, Cramer *V*=0.389; *P*=.009; Figure 4).

### Dropouts

Two participants in the assisted onboarding actively terminated the study prematurely after 14 days and 16 days. Reasons for dropout were a broken phone and poor health status. Another dropout occurred in the video group after 2 weeks. The participant mentioned feeling that they are not useful in contributing to the study. Baseline characteristics were comparable between participants who actively dropped out and those retained.

### Time-to-Account Creation

The time between account creation and the onboarding date was calculated for all participants who downloaded the application in the standard and video onboarding groups. The mean time to download was 5.61 (SD 6) days, with a range of 0 to 23 days. In total, 36% (12/33) of participants downloaded the application before the first reminder (at the end of the first week) and 64% (21/33) after the first reminder. There was no significant difference observed between the standard and the video group (*P*=.63).

### Engagement: Minutes, Log-Ins, and Survival

**Figure 5:**
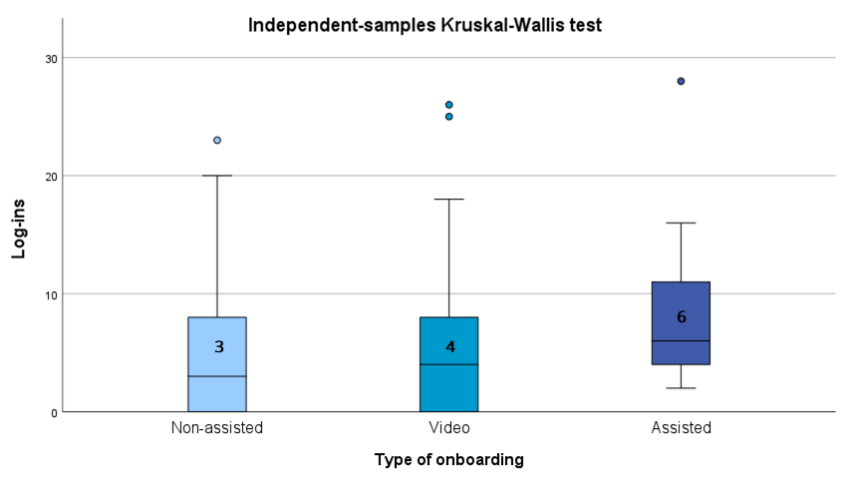
Median number of log-ins per onboarding group.

In total, 96 screenshots of usability data were expected to be provided by participants. About 25% (12/48) of participants submitted usability data; however, these data were mostly incomplete (eg, only providing the minutes spent on the application for a single day). Out of the usability data obtained in minutes, participants used the application for 14 to 68 minutes during a week. Cumulative log-ins were passively collected. The number of log-ins for the duration of the study ranged from 0 to 28, with an overall mean of 7 (SD 7.68). A Kruskal-Wallis test showed no significant differences between the 3 groups (Kruskal-Wallis test *df*=2, median 3, IQR 8 vs median 4, IQR 11vs median 6, IQR 8; *P*=.18), as displayed in Figure 5. As no group differences were observed for onboarding type, we additionally ran a linear regression including the available covariates (sex, age, depression, anxiety, and opioid use) to examine their influence on engagement (number of log-ins during the 1-month period). Anxiety was a significant predictor of log-ins (β=−5.148, 95% CI −10.2 to −0.09; *P*=.046).

**Figure 6:**
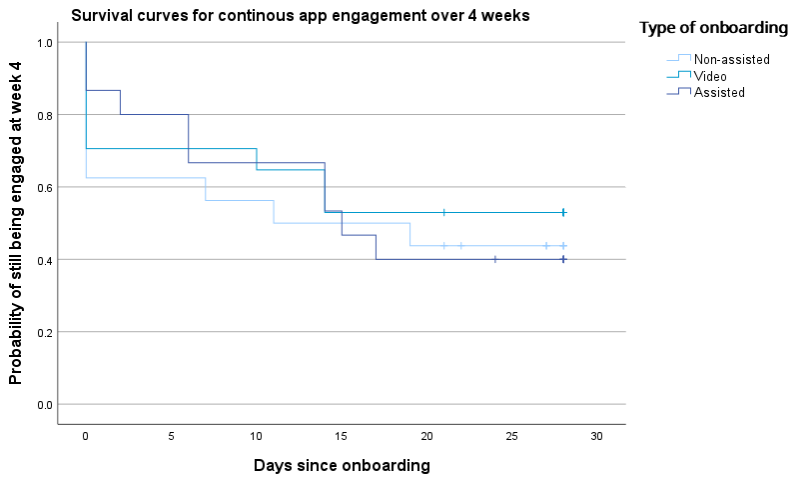
Survival curves for the probability of still engaging with the application in the last week of the study per onboarding type.

For the Kaplan-Meier survival analysis, we examined how many participants were still using the application during the final week of the 4-week study period. Time-to-event was defined as the number of days between onboarding and the participant’s last log-in. An event was considered to have occurred if a participant did not use the application during the final study week, meaning that their last log-in occurred before week 4. A log-rank (Mantel-Cox) test revealed no significant difference in survival distributions between the onboarding groups (*χ*^2^_2_=0.4; *P*=.82; Table 2). The overall survival rate was 46% (22/48), with a mean survival time of 15.9 days. Among the groups, the video-onboarded participants showed the highest probability of continued application use, with a survival rate of 53% (9/17) and a mean survival time of 17.1 (SE 3.0) days (Figure 6).

**Table 2:** Survival data stratified by onboarding type.

Type of onboarding	Participants, n (%)	Events, n (%)	Survival rate (%)	Survival duration (days), mean (SE)
Standard	16 (33)	9 (56)	44	14.6 (3.2)
Video	17 (35)	8 (47)	53	17.1 (3.0)
Assisted	15 (31)	9 (60)	40	16.1 (2.8)

### Adherence

Out of the 48 participants, only 13 (27%) were adherent with at least 1 connection per week for 4 weeks. Adherence was not influenced by onboarding group membership.

### Participant Satisfaction: SUS

About 17% (8/48) of participants answered the SUS survey, leading to an overall score of 70.31. This score suggests a “good” usability level, as SUS scores typically range as follows: >78.8 (excellent usability), 70-85= (good usability), 50-69= (acceptable usability), and <51.6 =(poor usability) [31]. Only 50% (3/6) of participants provided feedback on their satisfaction with the onboarding type they received (standard and assisted groups); they were all satisfied and mentioned that the instructions that they received were clear and sufficient. Suggestions to improve the application by participants included fixing technical bugs, adding other language options, expanding content, integrating goal-setting features, enabling chat options with peers and HCPs, improving the design, providing links to scientific evidence and statistics about the disease, adding subtitles to videos, including a place to take notes, incorporating exercises or advice from ergotherapists, and implementing nudging strategies.

## Discussion

### Principal Findings

In this study, we found that in-person assisted onboarding significantly increased the adoption of a newly developed self-management application among patients with chronic musculoskeletal pain. While 81% (39/48) of participants downloaded the application and 75% (36/48) used it at least once (ie, adopted it), sustained use declined notably after approximately 2 weeks, with only 46% (22/48) still using the application by the fourth week. Mean and median log-in frequencies were highest in the assisted onboarding group and progressively lower with decreasing levels of support. However, onboarding type did not significantly influence overall engagement, as measured by median log-ins throughout the study period, nor adherence to regular use. The only significant predictive factor for app engagement we found was anxiety. Usability and satisfaction with onboarding were also assessed; however, due to the limited number of participants who provided detailed usability metrics in minutes, no statistical comparisons between onboarding groups could be performed for these outcomes.

Assisted in-person onboarding was effective for the adoption of the POCOS application and facilitated the timely initiation of application use, in contrast to the other two groups where account creation generally occurred a week later, aligning with the first reminder they received. Studies using health applications should consider implementing assisted onboarding to guarantee a seamless start to the study. On average, participants logged into the application 7 times during the study, and the time spent using the app ranged from 14 to 68 minutes per week. Our results regarding adoption and engagement are comparable to those reported in other mHealth studies in populations with chronic pain [32,33]. For example, in one study evaluating a chronic pain management app, 45% of participants remained engaged after 1 month [33], while in another study, 39% of participants completed the 3-month self-management program of a chronic pain app [32]. In addition, mHealth studies primarily focusing on depression and anxiety [7-9,34] may also be considered, as these conditions are highly prevalent among patients with chronic pain. Compared to our study, fewer participants downloaded the app in these studies, which may be explained by the fact that one group in our study received tailored support. In one of these studies, higher baseline depression and anxiety scores were associated with lower app use; similarly, anxiety was negatively associated with engagement in our study. This relationship warrants further investigation.

However, overall adherence in these studies was higher than in our study. This difference may be partly explained by the fact that patients with chronic pain often experience, in addition to depression and anxiety, other comorbidities and persistent pain, which may further complicate engagement and adherence. In particular, our sample included patients with complex chronic pain conditions, all of whom were refractory to treatment. Besides measuring engagement, it is important to define what meaningful engagement is [35]. For example, more log-ins do not automatically mean that a patient engaged more with the application. To analyze this, the minutes spent on the application would need to be structurally captured. Moreover, meaningful engagement not only is time related but also can include engagement with certain parts of the application, regularity of use, or sustained use for a certain period. Another aspect is that patients’ symptoms might improve over time, and therefore, the application becomes less needed, which could be shown in less use [35]. Therefore, it is important to investigate which part or combination of the application is effective or how much time patients have to spend on the application to achieve an improvement of their symptoms when designing studies with therapeutic applications. A systematic review of mHealth interventions for chronic pain self-management reported study durations ranging from 4 to 24 weeks [36]. In comparison, the survival time observed in our study with the POCOS application was relatively short. If the effectiveness of the application is to be assessed in future studies, extending survival time by implementing strategies to foster user engagement will likely be necessary. The successful phone support that has been provided for participants who reached out for help with downloading and account registration suggests that remote but assisted onboarding could be another option to improve adoption. Future research should investigate whether this approach could be an option on a larger scale, as this would offer more flexibility for patients and HCPs than in-person assisted onboarding. It remains unclear why none of the participants in the standard onboarding group reached out to the research team for additional support to download and create an account, while in the video group, 4 participants reached out. We assume that after watching the video showing and explaining the application, participants from video group got more motivated and incentivized to use the application than the participants from the standard group.

Application engagement was consistent across onboarding groups, likely because the application was user-friendly and the user manual was clear and sufficient. The SUS score of 70.31 supports the hypothesis that the application has good usability. However, the score must be interpreted with care due to the limited response rate. Feedback from participants showed that there is room for improvement in the technical functionality and content of the application. This could be addressed with another round of cocreation and design workshops with patients, as well as input from other HCPs.

Although engagement strategies were considered during the application’s development, other factors may also play a role and warrant further investigation. For example, previous studies have identified several patient-level factors that negatively influence engagement with mHealth applications, including higher pain levels, depression, longer disease duration, male sex, and lower sociodemographic status or education level [20,37]. Participants in our cohort were characterized by high levels of pain and depression and had a long-standing, treatment-refractory condition, which may have negatively affected adherence and sustained engagement with the application. Patients with chronic pain are considered particularly challenging with regard to adherence compared to other patient groups. This is partly due to their long-term experience with limited symptom relief. The complexity and multimodal nature of their treatments result in a high treatment burden. Additionally, emotional factors such as depression, anxiety, and frustration are more prevalent in this population and can negatively affect motivation and confidence in managing treatment, further reducing adherence [38-40]. Studies investigating adherence to medication among patients with chronic pain have shown, for example, that patients often report taking their medication as prescribed, while in reality they self-adjust their intake. Medication nonadherence in this population is a known issue, although it varies across countries, ranging from 8% to 60%. A systematic review found that age, pain intensity, and the patient-caregiver relationship were negatively associated with adherence to analgesic medication in patients with chronic pain [38,41-43]. While there is extensive literature on medication adherence, less is known about adherence to mHealth interventions, particularly in populations with chronic pain. It is possible that behaviors or factors that negatively influence medication adherence may also translate into reduced adherence when using digital health applications. For example, a study investigating predictors of patient engagement with an app for chronic pain patients reported that those experiencing higher levels of pain showed better engagement with the app than patients whose pain was less severe [44]. Identifying more characteristics in patients with chronic musculoskeletal pain associated with higher application use could enable the development of patient profiles, recognizing that mHealth solutions may not be equally suitable for all individuals.

Beyond the limited return of SUS survey responses, participants provided minimal insight into their application use behavior (eg, minutes spent on the application). The active provision of usability data seems to be too demanding for participants and should be switched fully to passive collection, as has been done with the log-ins. To improve adherence, measures such as increased follow-up, support and monitoring by study personnel, or reimbursement for study tasks could be an option [45].

### Limitations

Although our study provided insights into the barriers of managing the onboarding process without assistance, there are some limitations. We did not track whether participants of group 2 watched the video or how often; however, we were able to track the views and downloads as a total. Therefore, we cannot say with certainty that every participant watched the video. However, on the basis of 17 views and 33 downloads, it is most likely that each of the 17 participants in group 2 had watched the video at least once. The application used in this study is still under development, which led to the occurrence of a technical bug during the 2-week period. The section with advice had disappeared for 3 patients, which might have demotivated some patients from using the application regularly. Although the usability of the application was assessed in this study, no conclusion can be drawn from the SUS score due to the small amount of feedback that has been obtained. A formal sample size calculation was not undertaken, as this study was designed as a feasibility study. Therefore, the sample size was based on practical considerations rather than on estimating or detecting an effect size. Therefore, this study had a small sample size and should be replicated with a larger cohort to assess the robustness and replicability of the findings. Future studies should also include more detailed patient characteristics and demographics to provide a more comprehensive understanding of the factors influencing adoption and engagement. In particular, variables such as depression and anxiety should be measured on a continuous scale rather than only as binary indicators, to capture the full range of symptom severity and their potential impact on engagement. Moreover, as patients were together undergoing a 2-week in-house multimodal chronic pain program before the study and commonly maintained contact beyond its completion, randomization was not feasible owing to a substantial risk of cross-group contamination.

### Conclusions

Onboarding without in-person assistance appears to potentially hinder the adoption of digital health applications, even among relatively younger users (mean age 45.7, SD 12.2 years). While these apps are designed to support remote access and use, HCPs should still consider offering guidance during the initial setup, particularly for downloading and registration.

## Data Availability

The datasets generated or analyzed during this study are available from the corresponding author on reasonable request.

## Appendix

Multimedia Appendix 1 User manual for the study and pain organizer and companion system (POCOS) app.

## Abbreviations

HCP: health care professional
mHealth: mobile health
POCOS: pain organizer and companion system
SUS: System Usability Scale
