# Supplementary material for: Impact of Different Onboarding Strategies on Low Adoption and Engagement With a Self-Monitoring and Management App for Chronic Musculoskeletal Pain: Prospective Study
Source: JMIR Mhealth Uhealth. 2026 Mar 30;14:e78827. doi: 10.2196/78827 (PMC13077279; doi:10.2196/78827)

Instructions de démarrage de POCOS : l'application d'autogestion pour la fibromyalgie post-virale

Rappel

- S'inscrire dans POCOS
- Utiliser POCOS au minimum 2-3x par semaine 15 minutes durant 4 semaines
- Mettre à jour ses données (health assessment) au moins 3 x par semaine
- Envoyer les captures d'écran (données d'utilisation) après 2 et 4 semaines
- Remplir le sondage de satisfaction à la fin de l'étude

Sécurité et protection des données

Les données sont collectées sur POCOS de manière anonyme, et sont stockées indépendamment des informations personnelles des utilisateurs. Les mots de passes sont cryptés. Les données sont exclusivement utilisées dans le but scientifique et médical d'améliorer le développement de POCOS et le processus thérapeutique ciblant la fibromyalgie et les syndromes chroniques post-viraux similaires.

Informations générales

POCOS doit être utilisé de manière régulière (minimum 2-3 fois par semaine, durant 4 semaines). À chaque utilisation, l'utilisateur est invité à remplir le questionnaire ainsi qu'à visualiser l'évolution de ses données et à s'engager dans des modules thérapeutiques (physio, modules éducatifs, exercices thérapeutiques…), durant au moins 15 minutes. Dans le cadre de l'étude, les utilisateurs seront contactés pour répondre à de courts questionnaires sur leur utilisation de POCOS. Ils seront également notifiés par email ou SMS à fréquence régulière durant 4 semaines sous forme de rappels à utiliser POCOS.

Relevé de données d'utilisation

Toutes les deux semaines (durant les 4 semaines), vous êtes tenus d'envoyer les données relatives à votre utilisation de POCOS à l'équipe de recherche. Pour ce faire, vous devez simplement vous rendre dans les paramètres de votre téléphone portable et prendre des captures d'écran des données d'utilisation de l'application pour les deux dernières semaines. Vous transmettrez ensuite ces captures d'écran à l'équipe de recherche. Les instructions détaillées sont fournies en annexe. Vous recevrez de l'assistance en cas de difficulté à effectuer cette opération.

Accès

1. Android : Si vous avez un téléphone mobile sous Android, veuillez installer l'application POCOS sur votre smartphone en cliquant sur le lien suivant ou en scannant le QR-code ci-dessous. Cela vous redirigera vers le fichier de téléchargement (.apk) :
   https://drive.google.com/file/d/1_dYpJE98k0MfB56HdbFYZexZmsCXDq7O/vie

QR-code 1: POCOS pour Android

1. iOS/Apple : Si vous avez un iPhone, veuillez d'abord télécharger l'application TestFlight depuis l'AppStore (QR-code 2 ci-dessous), puis ensuite utiliser ce lien : https://testflight.apple.com/join/SxF4z6d9 ou scanner le second QR-code (QR-code 3) pour télécharger POCOS (TestFlight permet l'utilisation d'applications test).

QR-code 2: TestFlight

QR-code 3: POCOS

Inscription

Inscrivez-vous ensuite en cliquant sur « Register » puis en complétant les champs. Vous allez alors recevoir un e-mail de confirmation avec un lien qui vous permettra de vous confirmer et de vous connecter.

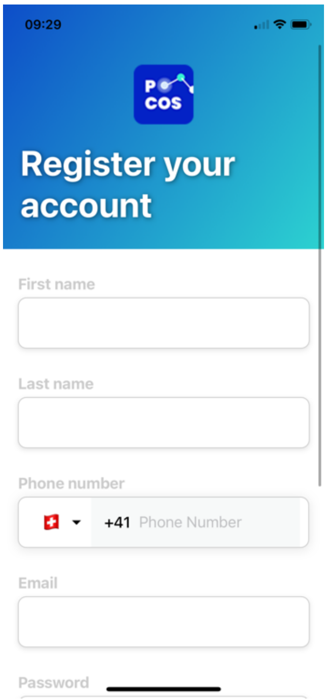

Connexion

Veuillez ensuite vous connecter à POCOS en cliquant sur « Login » après avoir rempli les champs *Email* et *Mot de passe.*

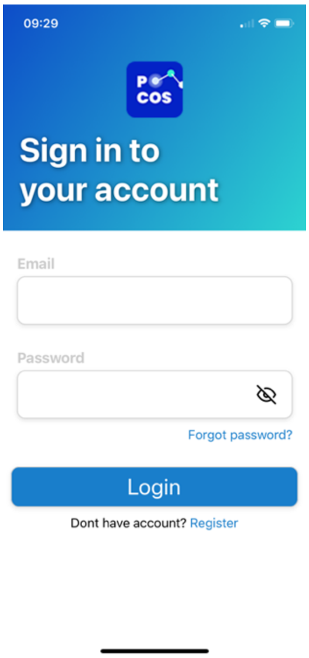

Bienvenue ! Première connexion

Bienvenue sur POCOS ! Lors de votre toute première connexion, quelques questions spécifiques vous seront posées. Merci d'y répondre et de compléter les données manquantes afin de pouvoir commencer à utiliser POCOS.

Comment allez-vous ?

La première section de POCOS s'intitule « État de santé » (à gauche dans la barre de menu). Ici, vous pouvez régulièrement entrer les données relatives à vos symptômes. Nous vous recommandons de le faire le plus souvent possible, au moins 3 fois par semaine.

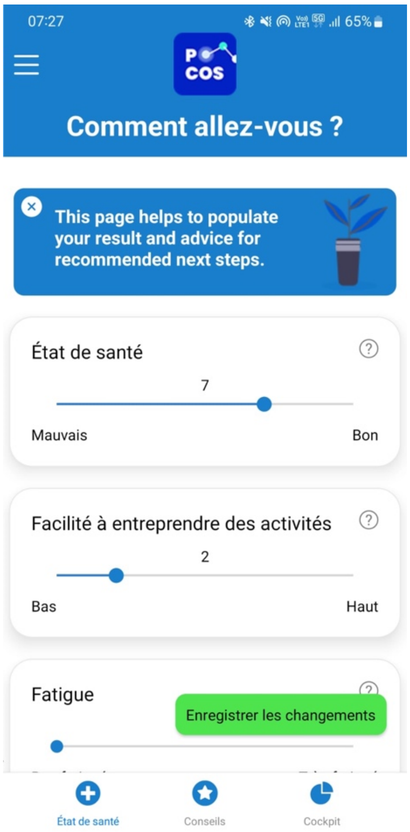

Vue d'ensemble

Cockpit (à droite dans le menu) est la seconde partie de POCOS, où vous pouvez suivre l'évolution de vos symptômes.

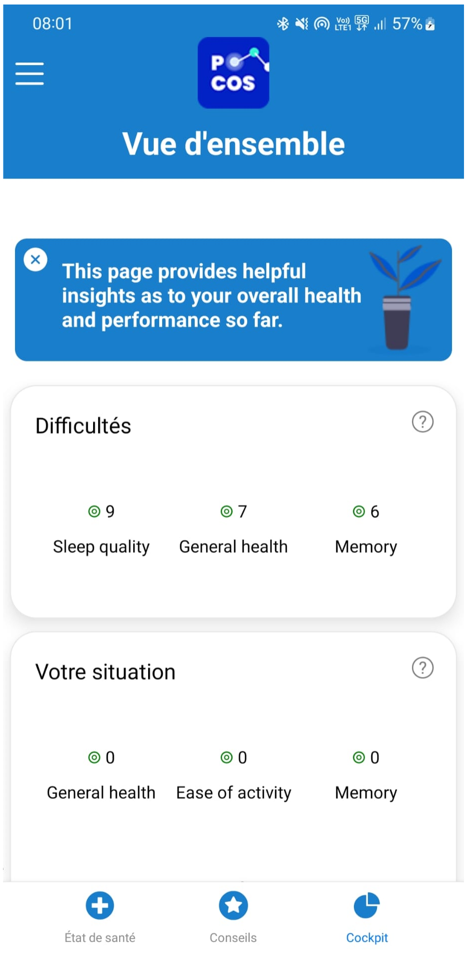

Quelques Conseils

Finalement, « Conseils » est la partie centrale de POCOS, où vous retrouverez respectivement :

- Des modules d’entrainement spécifiques à des symptômes ou à but éducatif pour vous aider au quotidien, et parfois avec des trucs & astuces pour gérer vos symptômes.
- Des modules de physiothérapie avec des exercices vidéo ciblés pour la réhabilitation.

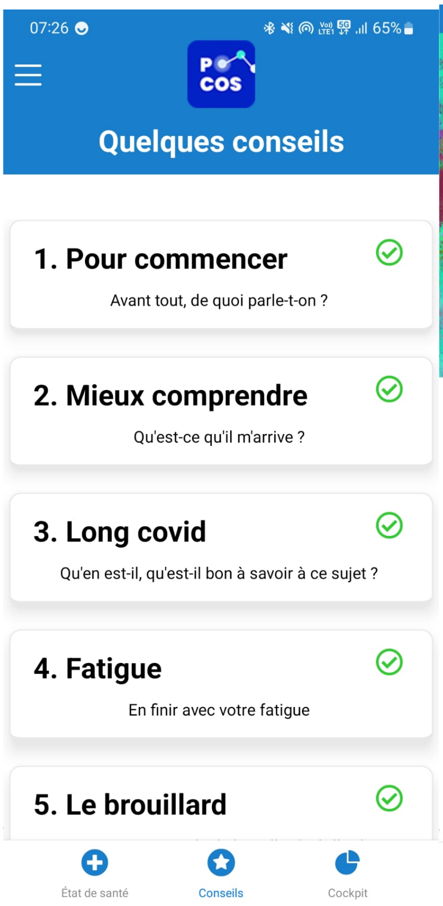

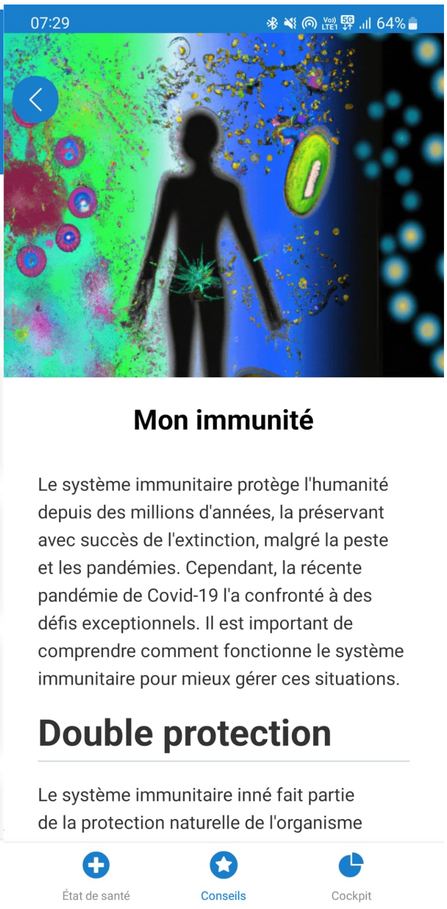

Contact

En cas de questions ou problèmes au sujet de POCOS et de son utilisation, veuillez contacter l’équipe de recherche à l’adresse suivante :

marc.blanchard@chuv.ch

Annexe

- Instructions pour l'envoi des données d'utilisation de POCOS à la fin de semaine 2 et 4 :
- Traduction des expressions/mots anglais dans POCOS

Traduction des expressions/mots anglais dans POCOS

ONBOARDING

Date of birth	Date de naissance
Height	Taille (cm)
Sex	Sexe
Country	Pays
Address	Adresse
Postal code	Code postal
Education	Niveau d'étude
Smoking	Fumeur/euse

Étude onboarding POCOS: instructions pour l'envoi des données d'utilisation

Pour les utilisateurs d'un smartphone Apple (iOS)

1. **Réglages**
2. **Temps d'écran**
3. **Voir toute l'activité**: vérifier que l'affichage corresponde à la semaine concernée
4. Sous « Les plus utilisées », ouvrez **POCOS** (si elle n'apparait pas dans le menu, cliquer sur Plus jusqu'à ce que vous la trouviez)
5. Prenez une capture d'écran (screenshot), voir raccourci du téléphone, en général, appuyer simultanément sur le bouton de démarrage et le bouton pour augmenter le volume
6. Envoyer la capture d'écran correspondante aux organisateurs de l'étude, soit par mail: marc.blanchard@chuv.ch, soit par téléphone au +41794266883 (whatsapp ou SMS)

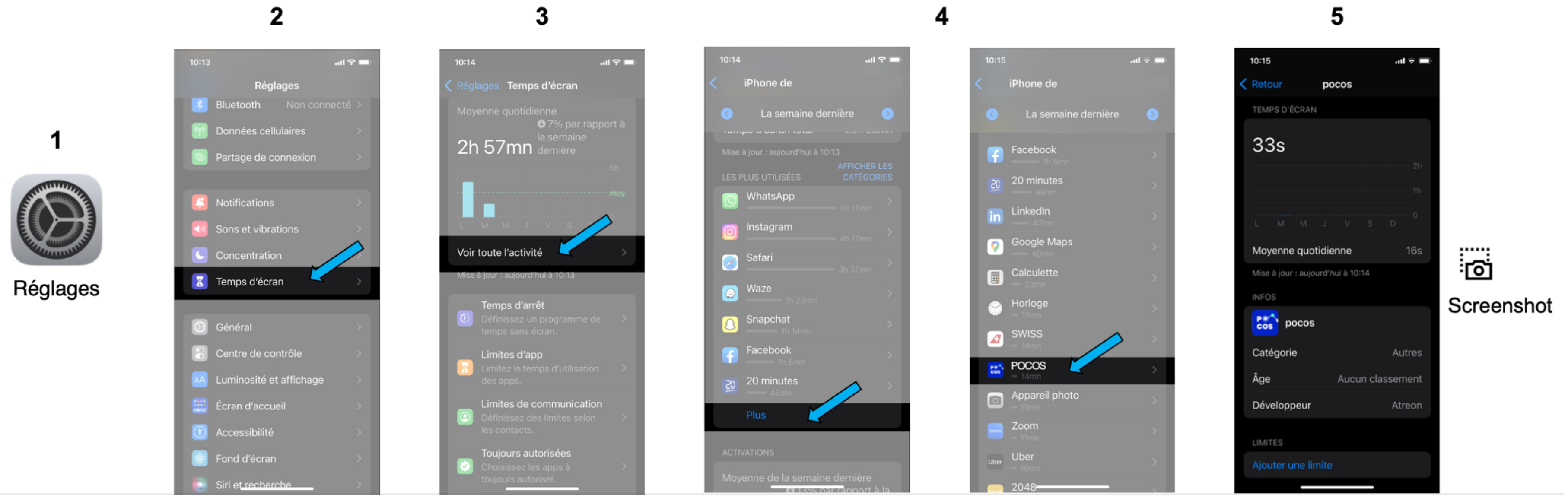

Étude onboarding POCOS: instructions pour l'envoi des données d'utilisation

Pour les utilisateurs d'un smartphone Android

1. **Paramètres**
2. **Applications**
3. Dans le menu défilant, chercher **POCOS** et ouvrir
4. **Temps d'écran**
5. Sélectionnez **Hebdomadaire** (si ce n'est pas déjà le cas) et vérifier que l'affichage corresponde à la semaine concernée
6. Prenez deux captures d'écran (screenshot), la première avec le temps d'écran, puis la seconde avec le nombre d'ouvertures (scroll vers le bas), voir raccourci du téléphone en général, appuyer simultanément sur le bouton de démarrage et le bouton pour baisser le volume
7. Envoyer la capture d'écran correspondante aux organisateurs de l'étude, soit par mail: marc.blanchard@chuv.ch, soit par téléphone au +41794266883 (whatsapp ou SMS)

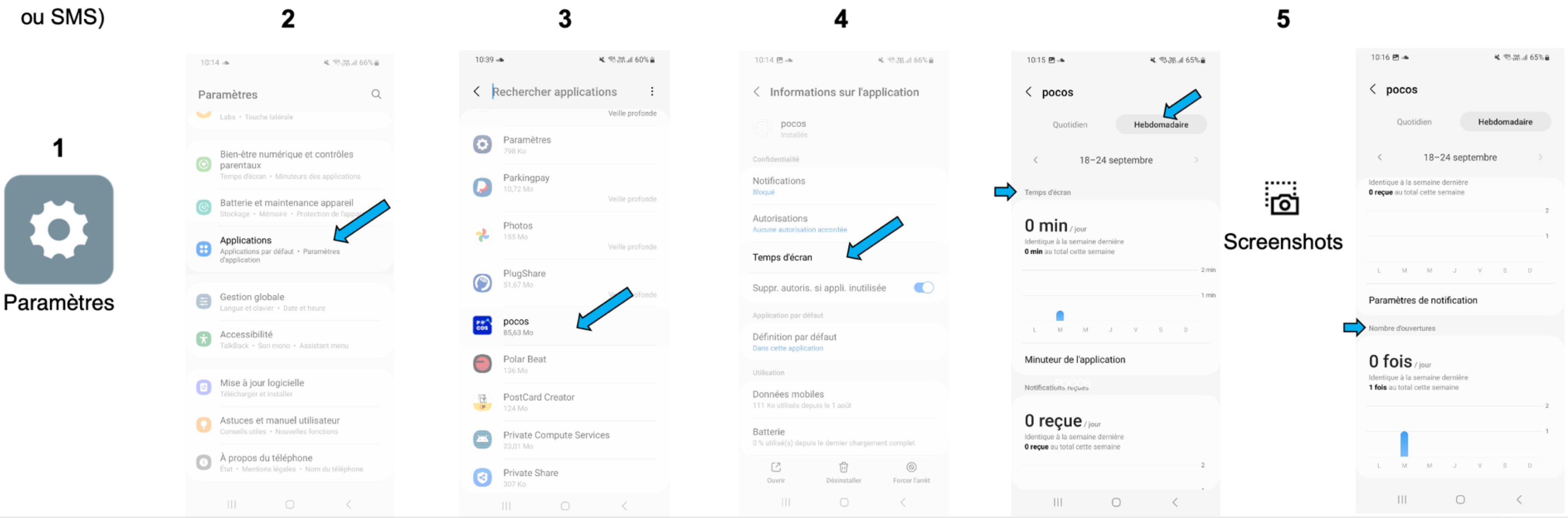

Supplement: Multimedia Appendix 1 [file mhealth_v14i1e78827_app1.pdf]
